# Heavy metals in playgrounds in Lublin (E Poland): sources, pollution levels and health risk

**DOI:** 10.1007/s11356-020-09375-y

**Published:** 2020-06-14

**Authors:** Wojciech Zgłobicki, Małgorzata Telecka, Sebastian Skupiński

**Affiliations:** Institute of Earth and Environmental Sciences, Maria Curie-Skłodowska University, Kraśnicka 2D, 20-718 Lublin, Poland

**Keywords:** Health risk, Heavy metals, Urban environment

## Abstract

In the paper, the pollution of playgrounds in Lublin with heavy metals was assessed. Since playgrounds are a place of activity of children—the most vulnerable group of city residents, identifying the degree of pollution and the related health hazards is particularly important. 50 playgrounds were investigated, and samples were collected from three types of places: soil in the playgrounds, soil beneath the swings and soil in the sandpits (a total of 91 samples); heavy metal levels were determined in the < 0.05-mm fraction. The mean heavy metal levels were Cd—4.7 mg kg^−1^, Cr—192.4 mg kg^−1^, Cu—16.3 mg kg^−1^, Hg—0.027 mg kg^−1^, Ni—12.7 mg kg^−1^, Pb—41 mg kg^−1^, and Zn—79.8 mg kg^−1^, and they were in the following order when normalised to the geochemical background: Cd > Cr > Zn > Pb > Hg > Ni > Cu. The highest mean levels occurred in the case of soil collected from beneath the swings (Cd, Cr, Ni and Zn) or soil in the playgrounds (Cu, Hg and Pb). Geochemical indices such as *I*_*geo*_ and *EF* indicate the lack of pollution or low level of pollution with Cu, Cr, Ni, Zn and Pb. Moderate or considerable pollution with Cd and, sporadically, with Hg occurred in some playgrounds. A significant ecological risk was found for all samples due to the presence of Cd and, for about 20% of the samples, the concentration of Hg. In all the cases analysed, the values of health hazard indices (*HI*, *CR*) for children are very low and well below the hazard threshold for each element.

## Introduction

The occurrence of increased heavy metal levels is one of the significant threats to the human environment and health in urban areas (Wong et al. 2006; Guney et al. 2010; Charlesworth et al. 2011; Xia et al. 2011). Their levels in the environment are determined by natural factors and anthropogenic supply, i.e. industrial emissions, motor vehicle traffic, fuel combustion (Kabata-Pendias and Pendias 1999; Pasieczna 2003). Metals discharged into the atmosphere gradually accumulate in the soil. Wherever the soil is not protected by vegetation, heavy metals can be re-suspended and can occur at low heights—in human living space. This creates the risk of toxic elements entering human bodies, which can pose a threat to their health (Aelion et al. 2008; Zheng et al. 2010; Charlesworth et al. 2011; Gope et al. 2017). Playgrounds are a special kind of space in this respect because the intensive resuspension of heavy metals accumulated in the soil occurs as a result of children’s activity (wearing away of the topsoil), while the children staying in the playground are particularly exposed to the negative effect of toxic metals. Children are active close to the land surface and absorb the soil dust and the metals it contains by inhaling it or directly by ingesting it through the alimentary tract (Elom et al. 2013). In both cases, the fine dust fraction poses the greatest threat (Yamamoto et al. 2006). Higher concentrations of lead in dust on playground equipment comparing to soils where reported in Beijing by Peng et al. (2019). Therefore, it is important for persons using playgrounds to maintain personal hygiene, i.e. wash their hands.

The problem of the pollution of playgrounds has been taken up by researchers in various countries and cities, but few studies have been devoted to this topic, while the obtained results concerning the intensity of the threats vary. For example, De Miguel et al. (2007) studied heavy metal levels in 20 playgrounds in Madrid and found that increased As levels can be a health hazard. High levels of toxic elements occurred in the soils of playgrounds located in the service and industrial zones in Hong Kong (Wong and Mak 1997). Elom et al. (2013) examined samples from 29 playgrounds in NE England for the presence of As and Pb and found that the concentrations of these elements could be a health hazard. Similar conclusions were reached by Kicińska et al. (2017) and Kicińska (2018) who analysed the pollution of sandpits with heavy metals in health resorts in southern Poland. Although they found a considerable level of pollution with these elements, in particular As and Cr, it was not high enough to pose a threat to health. Identifying the sources of pollution in playgrounds is a different matter. Research indicates that pollution originates from different sources: traffic, local and regional atmospheric deposition, natural sources (Jin et al. 2019).

The study objective was to assess heavy metal levels in the soil of playgrounds and the health hazard to children staying there. The chosen metals—Cd, Cr, Cu, Hg, Ni, Pb and Zn—are commonly regarded as indices of environmental pollution caused by human activity in urban environments (De Miguel et al. 1997; Kabata-Pendias and Pendias 1999; Charlesworth et al. 2011). Studies on heavy metal levels in playgrounds and the health aspects had not been conducted in eastern Poland thus far.

## Materials and methods

Lublin is a medium-sized city located in the north-western part of the Lublin Upland, eastern Poland (51° 08′ 23.31″–51° 17′ 47.61″N, 22° 27′ 15.41″–22° 40′ 24.75″ E). It covers an area of about 150 km^2^ and is inhabited by 350 thousand people. Lublin is now an academic, administrative and service centre. Small industrial areas are located in the eastern part of the city, and the influence of industrial emissions is spatially restricted due to the predominance of westerly winds. At present, not very large food, pharmaceutical, mechanical, chemical and construction industry enterprises operate in Lublin. Studies indicate a relatively low level of pollution of soils in the city with trace metals (Pasieczna 2003; Plak et al. 2010). The studies were primarily focused on the influence of motor vehicle traffic on the geochemistry of soils: samples were collected in the vicinity of streets. A not very high and decreasing level of street dust pollution with heavy metals was also found (Zgłobicki et al. 2018, 2019).

**Fig. 1:**
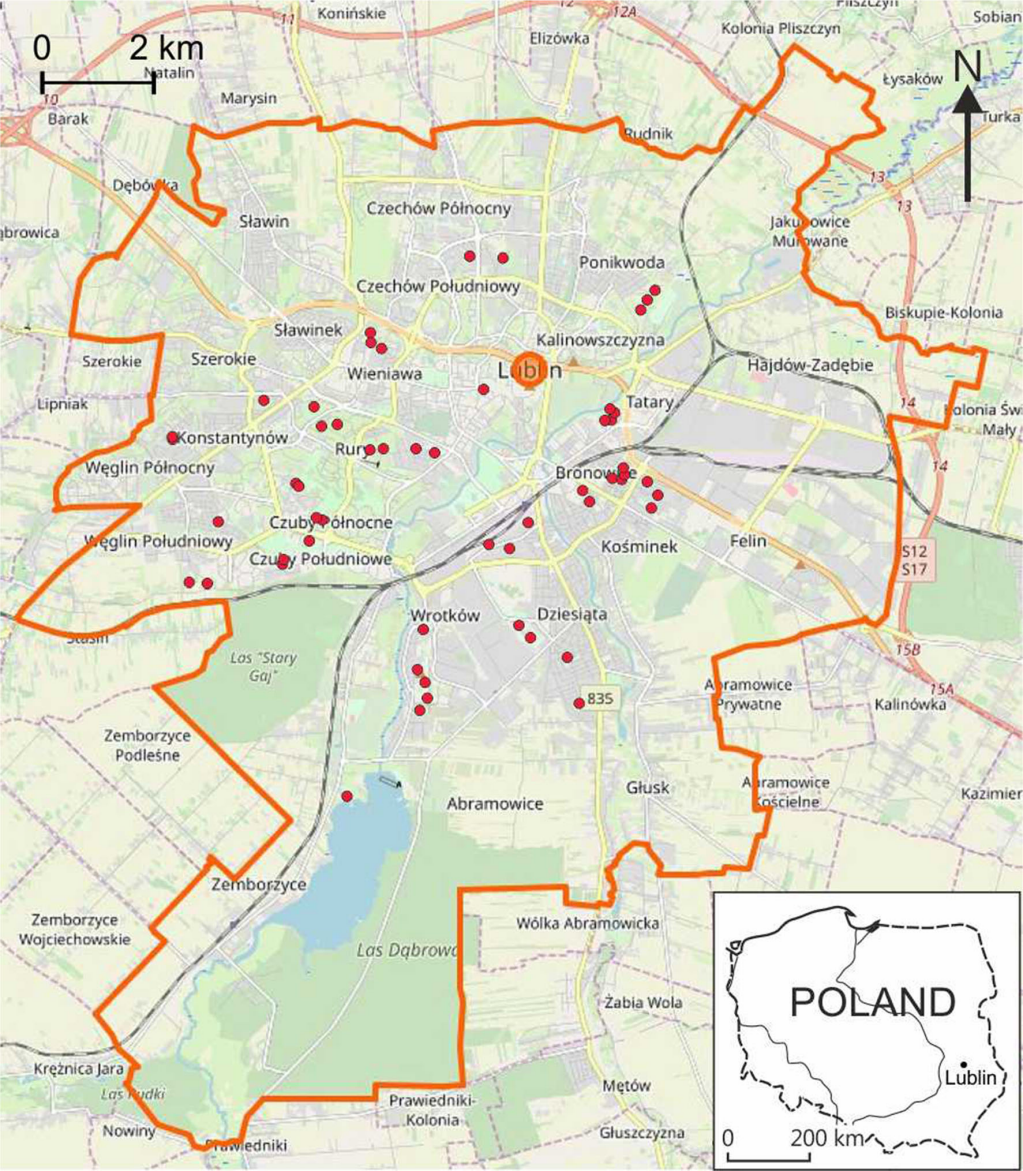
Location of the playgrounds under study (background map source: Open Street Map)

**Fig. 2:**
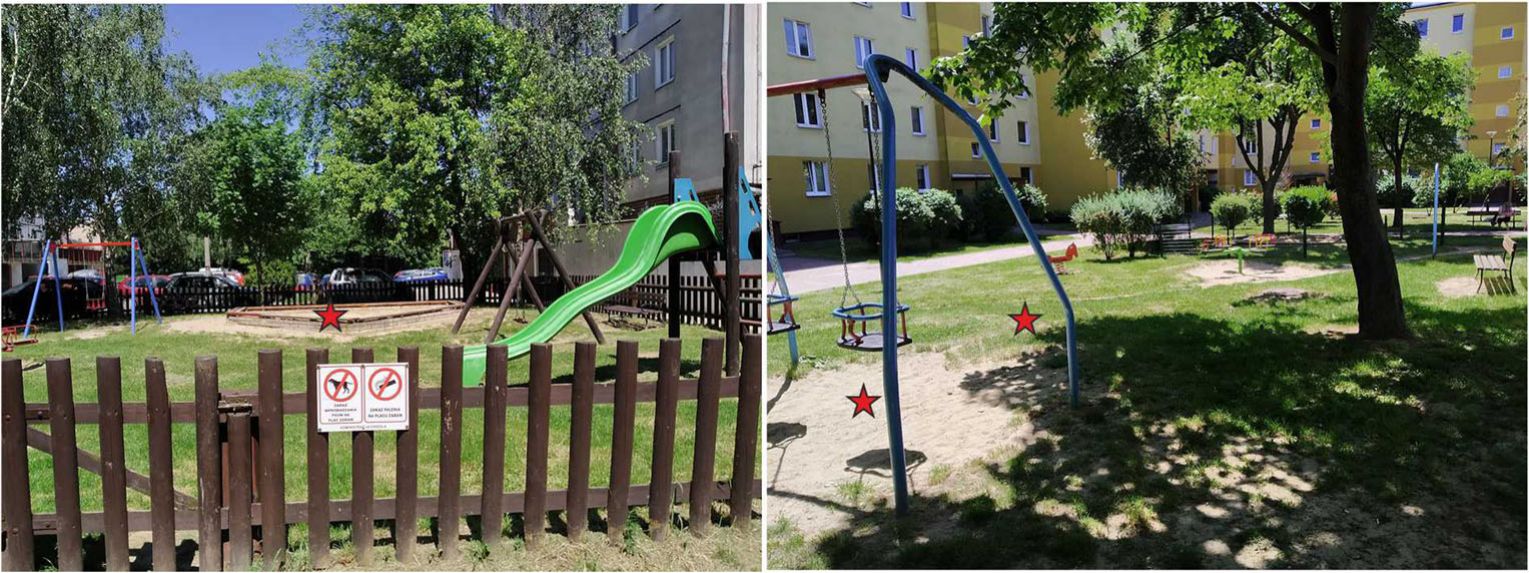
An example of sample collection points within a playground

The samples were collected within 50 playgrounds located in different parts of the city (Fig. 1). The locations were varied: inside housing estates sheltered by residential buildings; in close proximity to roads with a high intensity of traffic; industrial zones; recreational areas. The selected playgrounds differed in terms of facilities (swings, sandpits, ladders, tennis tables, chess tables etc.) as well as kind of surface (lawn, sand, synthetic flooring, hard surface) (Fig. 2).

The soil and sand samples were collected on days without precipitation from late spring until early autumn 2018 and 2019. The samples were obtained with a spade at various sites within the playgrounds. Material from three types of sites was analysed: (a) soil in playgrounds; (b) soil beneath swings; (c) soil in sandpits. Thus, heavy metal levels in the material that is most frequently re-suspended and with which children are in direct contact were analysed. Since all three types of sites did not occur in some playgrounds, the number of samples collected differed and, therefore, 44 soil samples from the playgrounds, 23 soil samples from beneath the swings and 24 soil samples from the sandpits were examined.

The samples were collected with a spade up to the depth of 5 cm at 5 to 7 points. In the case of soil sampling, exposed places without a vegetation cover were preferred. All portions of soil and sand from one type of site were joined together and packed into plastic bags. The sample weight after drying ranged from 1 to 2 kg. After being transported to the laboratory, the samples were stored in a warm place for 2 to 5 days for initial drying. In the subsequent stage, the samples were placed in porcelain evaporators and, if necessary, larger pieces of plants were removed. The drying in a laboratory dryer lasted for at least 6 h, at 105 °C. Analyses of heavy metals were carried out using the Epsilon 5 device from Panalytical and the ED-XRF method as well as the calibration curve method. The content of the analysed metals was determined in the <0.05-mm fraction. Samples for elementary analyses were prepared in the form of pressed tablets. Metal content was measured three times in each sample, and the mean of the measured values was accepted as the final result. The accuracy of the method was verified using reference material NCS DC 73385. The measurement error varied from 3 to 5%. The measurement results for 10 samples of soils collected in the immediate vicinity of the city and not subject to direct anthropogenic impact were used to determine the natural levels of metals in the environment (geochemical background).

Mercury was determined using the AMA-254 analyser. The measurements were carried out several times for each sample, and the final result was the mean of all the measured values. The weights for individual determinations (0.1–0.5 g) were selected so that the greatest sensitivity scope of the device was used.

Descriptive statistics (average, minimum, maximum, coefficient of variation) were used to assess the variation of heavy metal concentration in the soil and sandpits. In order to determine the degree of pollution, the following geochemical indices were calculated: (i) geoaccumulation index (*I*_*geo*_), (ii) enrichment factor (*EF*), (iii) index of ecological risk (*Er*_i_), and (iv) potential ecological risk index (*RI*) (Müller 1979; Håkanson 1980; Ergin et al. 1991; Zhao et al. 2015).

The geoaccumulation index is defined as follows:

$$ {\mathrm{I}}_{\mathrm{geo}}={\log}_2\left(\raisebox{1ex}{${\mathrm{C}}_{\mathrm{n}}$}\!\left/ \!\raisebox{-1ex}{$1.5{\mathrm{C}}_{\mathrm{ref}}$}\right.\right) $$

*C*_*n*_ denotes the concentration of the metal, and *C*_*ref*_ value of the background. Six classes of pollution are distinguished according to Müller (1979): (1) unpolluted (I_geo_ < 0), (2) unpolluted to moderately polluted (0–1), (3) moderately polluted (1–2), (4) moderately to strongly polluted (2–3), (5) highly polluted (3–4), (6) highly to extremely polluted (4–5), (7) extremely polluted (> 5).

Enrichment factor is calculated with the following formula:

$$ EF=\frac{C_n/{C}_{ref}}{B_n/{B}_{ref}} $$

*C*_*n*_—metal concentration in a sample, *C*_*ref*_—background value for the metal, *B*_*n*_—reference metal concentration in sample (in this study Mn was used as a reference element), *B*_*ref*_—background value for reference metal. Ergin et al. (1991) distinguish five of pollution: (1) minimal (EF < 2), (2) moderate (2–5), (3) significant (5–20), (4) very high (20–40), (5) extreme (> 40).

Index of ecological risk factor is defined by the following formula:

$$ {Er}_i={T}_i\times \frac{C_n}{C_{ref}} $$

T_i_—toxic-response factor for the metal. Håkanson (1980) provides the following values for T_i_: Cd—30, Cr—2, Cu—5, Ni—5, Pb—5, Zn—1. Five categories of pollution are distinguished: (1) low (< 40), (2) moderate (40–80), (3) considerable (80–160), (4) high (160–320), (5) very high (> 320).

Potential ecological risk (*RI*) is defined as the sum of ecological risk factors indexes (*Er*_*i*_) for specific metals in a sample. Håkanson (1980) distinguished four categories of risk: (1) low (< 150), (2) moderate (150–300), (3) considerable (300–600), (4) high (> 600).

The health hazard assessments combine the volume of environmental pollution with the probability of the toxic effect on people. The study uses the hazard index (*HI*) and cancer risk (*CR*) for a quantitative description of non-carcinogenic and carcinogenic risks related to the children’s exposure to heavy metals within playgrounds (US EPA 2001, 2005). These models are commonly used in studies on the hazard posed, for example, by heavy metals in street dust or in the soil in playgrounds (Jiang et al. 2017; Kicińska et al. 2017; Kicińska 2018; Pawełczyk et al. 2018; Safiur Rahman et al. 2019).

In the case of persons staying in a given area, there are three paths of direct contact with contaminants in soil and sand: (i) inhalation described with the parameter *D*_*inh*_, (ii) skin contact (*D*_*derm*_) and (iii) consumption (*D*_*ing*_). The exposure dose equations have the following form (US EPA 2005, 2011):

$$ {\displaystyle \begin{array}{c}{D}_{inh}=C\times \frac{ inh R\times EF\times ED\ }{PEF\times BW\times AT}\\ {}{D}_{derm}=C\times \frac{SL\times SA\times ABS\times EF\times ED\ }{BW\times AT}\times {10}^{-6}\\ {}{D}_{ing}=C\times \frac{ ing R\times EF\times ED\ }{BW\times AT}\times {10}^{-6}\end{array}} $$

where *C*—concentration of the element, *ingR*—ingestion rate dependent on the age, *inhR*—inhalation rate dependent on the age, *EF*—frequency of exposure to pollutants, *ED*—exposure duration, *SA*—skin area exposed to pollutants, *SL*—skin adherence factor, *ABS*—dermal absorption factor, *PEF*—particle emission factor, *BW*—average body weight, *AT*—averaging time in days (*AT* = *ED*·365) (Table 1).

**Table 1:** Exposure parameters for the health risk assessment for children (6 years old)

Parameter	Value	Unit
*ingR* (US EPA 2001)	200	mg day^−1^
*inhR* (Van den Berg 1995)	7.6	m^3^ day^−1^
*EF* (Safiur Rahman et al. 2019)	230	day year^−1^
*ED* (US EPA 2001)	6	years
*SA* (US EPA 2001)	2800	cm^2^
*SL* (Safiur Rahman et al. 2019)	0.2	mg cm^−2^ days^−1^
*ABS* (US DOE, 2004)	0.001	–
*PEF* (US EPA 2001)	1,360,000,000	m^3^ kg^−1^
*BW* (US EPA 1989)	15	kg
*AT1* (non-carcinogenic) (Kicińska 2018)	2190	days
*AT2* (carcinogenic) (Kicińska 2018, Safiur Rahman et al. 2019)	25,550	days

The hazard index (*HI*) is the sum total of hazards posed by the possible forms of pollutant absorption. It is calculated using the following equation:

$$ HI={\left(\frac{Di}{RfD}\right)}_{ing}+{\left(\frac{Di}{RfD}\right)}_{inh}+{\left(\frac{Di}{RfD}\right)}_{derm} $$

*Di* is the dose of element *i*, and *RfD* is the reference dose of the element. The proportions in the equation refer to non-carcinogenic effects. The probability of the occurrence of effects harmful to life is very low for *HI* < 1, while the occurrence of negative effects is probable for *HI* > 1 (US EPA 2005). In the case of *HI* > 4, the hazard is regarded as high (Pawełczyk et al. 2018; Safiur Rahman et al. 2019). For carcinogenic metals (As, Cd, Cr, Ni etc.), the lifetime average daily dose (*LADD*) is expressed with the following equation:

$$ LADD=C\times \frac{EF}{AT\times PEF}\times \left(\frac{CRchild\times EDchild}{BWchild}+\frac{CRadult\times EDadult}{BWadult}\right) $$

The equation takes into account the contaminants ingested by people or absorbed by inhaling polluted air, through skin contact with contaminated soil etc. in a specific time frame. For children, the equation takes the form:

$$ LADD=C\times \frac{EF}{AT\times PEF}\times \frac{CRchild\times EDchild}{BWchild} $$

For carcinogenic substances, i.e. As, Cd, Cr, Ni etc., *LADD* values are multiplied by an appropriate slope factor (*SF*) (Table 2), which enables the calculation of the cancer risk range (*CR*) (US EPA 2011)

$$ CR= SF\times LADD $$

**Table 2:** The reference dose and slope factor of metals (carcinogenic elements are marked in italic)

	Cd^1^	Cr^1^	Cu^1^	Hg^2^	Ni^1^	Pb^1^	Zn^1^
*RfD*_*ing*_	*0.001*	*0.003*	0.04	*0.0003*	*0.02*	0.0035	0.3
*RfD*_*inh*_	*0.001*	*0.0286*	0.042	*0.0000857*	*0.0206*	0.00352	0.3
*RfD*_*derm*_	*0.00001*	*0.00006*	0.012	*0.000021*	*0.0054*	0.000525	0.06
*SF*_*ing*_	*–*	*–*	–	*–*	*–*	–	–
*SF*_*inh*_	*6.3*	*42*	–	*–*	*0.84*	–	–
*SF*_*derm*_	–	–	–	*–*	*–*	–	–

^1^Safiur Rahman et al. 2019, ^2^Wang et al. 2019

Concentrations of metals can be regarded as hazardous to health when the cancer risk ranges (*CR*) are greater than 10^−6^ or 10^−4^ (the lower risk level that would be universally accepted has not been determined yet) (US EPA 1989; Pawełczyk et al. 2018; Safiur Rahman et al. 2019).

The cancer risk range *CR* is calculated for many substances as a sum of the risk for each of the metals studied:

$$ {CR}_{tot}={\sum}_{i=1}^n{CR}_i $$

where *CR*_*i*_ is a partial risk calculated for element *i*, and *n* is the number of elements analysed (Pawełczyk et al. 2018). In this case, the risk threshold is the same as for the cancer risk range calculated for a single element.

Principal Component Analysis (PCA) and Cluster Analysis (CA) were used to analyse the correlations between metal concentrations, which makes it possible to indicate the potential sources of their origin (Han et al. 2006; Tokalıoğlu and Kartal 2006). The PCA method is used to generate substitute parameters that encompass groups of output parameters with a similar statistical variability. The Cluster Analysis (CA) method that was also used enables the geometrical grouping of data in clusters with similar coordinates (in this case the coordinates encompass concentrations of metals). In this study, the minimum variance method was used (Ward’s method). Data normalised to the geochemical background were used in the cluster analysis. The normalisation of data enables the grouping of elements whose content levels are similar to the natural levels and elements whose levels exceed the geochemical background. The lack of normalisation to the local geochemical background can lead to mistaken conclusions when, for example, metals naturally occurring in high concentrations are grouped with metals whose concentrations are high as a result of human activity.

## Results

The mean heavy metal levels, normalised to the geochemical background, were in the following order for the samples analysed: Cd > Cr > Zn > Pb > Hg > Ni > Cu. Cd was the element with the highest degree of enrichment. Its mean level in playgrounds was more than five times as high as in natural soil. The maximum level was nearly 10 times as high. Slightly lower enrichment rates were found for Cr: the mean was about 3 and the maximum was over 9. The mean Pb and Zn levels were less than twice as high as the natural levels; the maximum levels were 9 and 11 times as high, respectively. Cu, Hg and Ni concentrations were usually close to the levels in soil not affected by human impact. The maximum rates of enrichment of the individual Cu and Ni samples were 2.5–3.2. Hg was the element whose levels exceeded the natural levels the most in individual samples: the maximum level was 26 times as high, and the level in two samples was 15 times as high. A high variation of concentrations occurred for Zn, Pb and Cu, medium variation—for Cr and Ni, and a low variation—for Cd (Table 3).

**Table 3:** Content of heavy metals in playgrounds (< 50 μm fraction) in Lublin (mg kg^−1^)

	Cd	Cr	Cu	Hg	Ni	Pb	Zn
Mean value	4.7	192.4	16.3	0.027	12.7	41.0	79.8
Median	4.5	201.3	15.5	0.021	12.0	38.2	78.3
Min. value	3.0	71.9	1.3	0.005	3.8	11.0	9.1
Max. value	8.8	566.6	60.4	0.54	31.7	191.2	476.1
Standard deviation	1.1	81.5	10.6	0.090	4.5	29.8	72.6
Variation coefficient (VC) (%)	23	42	65	300	35	72	90
Standard error	0.11	8.54	1.11	0.009	0.47	3.12	7.61
Mean value in street dust^1^	5.5	112.0	120.6	0.39^2^	17.1	46.6	296.2
Geochemical background	0.8	60.0	17.8	0.019	12.4	21.4	41.3
Limit values for soils of urbanised areas^3^	4	150	150	2	100	100	300

^1^Zgłobicki et al. 2019 (content in < 50-μm fraction), ^2^unpublished data, ^3^Dz.U.02.165.1359 2002

The average metal contents for Cd and Cr were slightly higher than the allowable concentrations for urban soils. For the remaining elements, in particular Cu, Hg and Ni, they were clearly lower than allowed (Table 3). They were exceeded by the maximum content in the case of Cr (3.7 times), Cd (2.2), Pb (1.9) and Zn (1.6).

The highest mean concentrations occurred in samples collected from beneath the swings (Cd, Cr, Ni and Zn) and from the soil (Cu, Hg and Pb). The differences between the particular kinds of samples were not large, however (Table 4). Hg was the exception; its levels were twice as high in soil samples from the playgrounds as in samples from beneath the swings, and three times as high as in sandpits. The patterns were slightly different in the case of maximum values. The differences were clearly greater here (Table 5). The highest level of Hg in the soil was over seven times as high as the maximum level in the sandpits. The highest Cr level was found in one sandpit; it was similar in the case of Ni. The highest levels of Cu, Hg, Pb and Zn occurred in the soil, and only in the case of Cd—beneath the swing.

**Table 4:** Mean heavy metal levels in various types of samples

Type of site	Cd	Cr	Cu	Hg	Ni	Pb	Zn
	mg kg^−1^						
Soil	4.6	192.2	19.2	0.045	11.7	47.4	90.3
Soil (under swing)	5.1	209.3	17.5	0.022	13.8	40.2	97.5
Sand box	4.5	177.8	11.4	0.015	13.6	32.0	52.6

**Table 5:** Maximum heavy metal levels in various types of samples

Type of site	Cd	Cr	Cu	Hg	Ni	Pb	Zn
	mg kg^−1^						
Soil	7.7	306.1	60.4	0.541	27.6	191.2	476.0
Soil (under swing)	8.8	448.1	56.0	0.272	27.5	168.2	421.0
Sand box	7.7	566.6	28.2	0.074	31.7	65.5	107.9

### Geoaccumulation index

There was no pollution with Cu, Ni and Zn, and no pollution with Pb and Cr for most of the samples. Medium pollution with Pb and Cr was found in a few samples (in the sandpits and beneath the swings). Pollution with Cd occurred in about 44% of the samples (soil in the playgrounds and beneath the swings). The pollution with Hg was at a medium level in two samples while high and very high pollution occurred in two and four soil samples, respectively (Table 6). Sample variation (variation coefficient) for *I*_*geo*_ was low for Cr, Cd and Ni, slightly higher for Cu, Pb and Zn, while the geoaccumulation index for Hg showed the highest variation, from -2.4 to 4.2 (Table 6).

**Table 6:** Descriptive statistics of geochemical indices

	Cd	Cr	Cu	Hg	Ni	Pb	Zn
Geoaccumulation index							
Mean	1.97	1.09	− 0.70	− 0.08	− 0.54	0.35	0.36
Median	1.90	1.16	− 0.78	− 0.45	− 0.62	0.25	0.33
Standard deviation	0.30	0.54	0.78	1.43	0.42	0.61	0.84
Variation coefficient	0.15	0.50	− 1.10	− 17.76	− 0.77	1.74	2.29
Share of polluted samples^1^	40.7%	7.7%	0%	8.8%	0%	4.4%	4.4%
Enrichment factor							
Mean	5.39	2.96	0.91	2.51	0.88	1.83	1.99
Median	5.10	3.04	0.83	1.08	0.89	1.64	1.73
Standard deviation	3.19	1.33	0.57	4.35	0.19	1.09	1.52
Variation coefficient	0.59	0.44	0.62	1.73	0.21	0.59	0.76
Share of polluted samples^1^	52.7%	3.3%	0%	9.9%	0%	3.3%	5.5%
Ecological risk factor							
Mean	181.28	6.88	5.26	111.17	5.36	10.74	2.31
Median	168.6	6.70	4.35	43.71	4.85	8.93	1.89
Standard deviation	41.94	2.73	2.99	194.68	1.81	7.00	1.76
Variation coefficient	0.23	0.39	0.56	1.75	0.33	0.65	0.76
Share of polluted samples^1^	91 (100%)	0 (0%)	0 (0%)	54 (59.3%)	0 (0%)	2 (2.2%)	0 (0%)

^1^share of samples (%), for which specific index is above the level “not polluted”

### Enrichment factor

The minimum enrichment was found for Cu and Ni, and for some Zn samples. A poor enrichment with Cr occurred for most samples; some samples also had a poor enrichment with Pb and Zn. A considerable pollution with Cd was found in 53% of the samples (soil in the playgrounds and beneath the swings). A considerable enrichment with Cr, Pb and Zn occurred in a few samples. In the case of one sample from a sandpit, a very strong enrichment with Cd was found. A strong pollution with Hg also occurred in nine samples, including two samples where it was very high.

### Ecological risk factor

Low levels of pollution were found for all elements except Cd. 35% of the samples showed a considerable pollution with Cd while a high level of pollution was found in 65% of the samples; a very high level of pollution occurred in one sample and a medium level in the remaining samples. A very high level of pollution with Hg occurred in eight samples, a high level—in three, and a considerable level—in 15 samples (Table 6).

### Potential ecological risk

Most of the samples (66%) had a moderate level of pollution (risk) and 33%—a considerable and high level. This concerned all three kinds of samples and resulted from the presence of toxic elements such as Cd and Hg. Cd showed a stable considerable and high level while Hg levels showed a considerable variation and fluctuated between a neutral and extreme level.

As was the case with absolute values, the soil in playgrounds had slightly higher geochemical indices than the sandpits (Table 7). For the enrichment factor, half of the soil samples from playground areas had a moderate level of pollution with metals such as Cd, Cr, Hg, Pb and Zn. The same level of this index for Cd and Cr occurred in soil samples from beneath the swings and from the sandpits. Half of the soil samples from beneath the swings had a high level of pollution with Cd in the case of the ecological risk index.

**Table 7:**
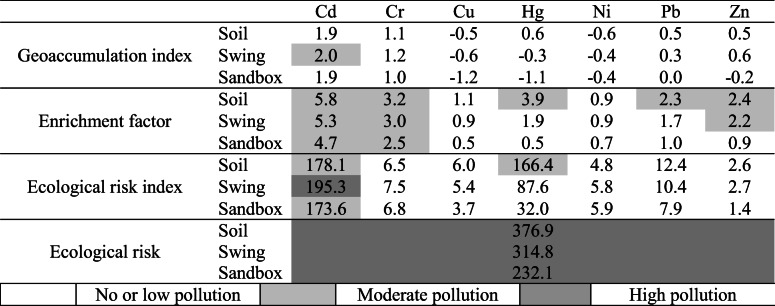
Geochemical indexes for different types of sites (median)

**Fig. 3:**
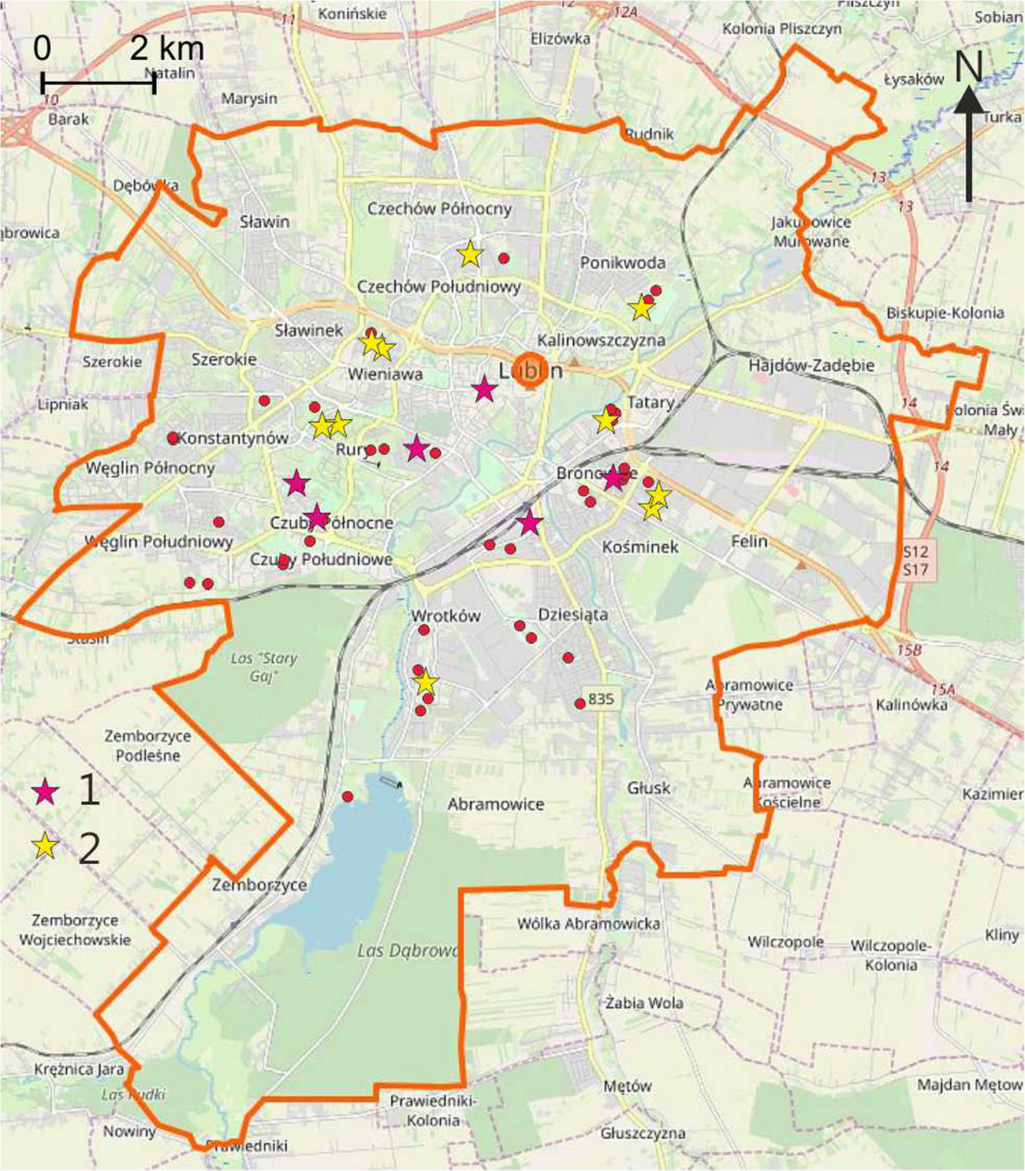
The most polluted playgrounds (based on the relation to the geochemical background and geochemical indices) 1—very high and high pollution, 2—high and considerable pollution (background map source: Open Street Map)

Playgrounds with the highest level of pollution occurred in the central part of the city, in the vicinity of the main streets and in the industrial and service district (Fig. 3).

### Statistical analysis

**Fig. 4:**
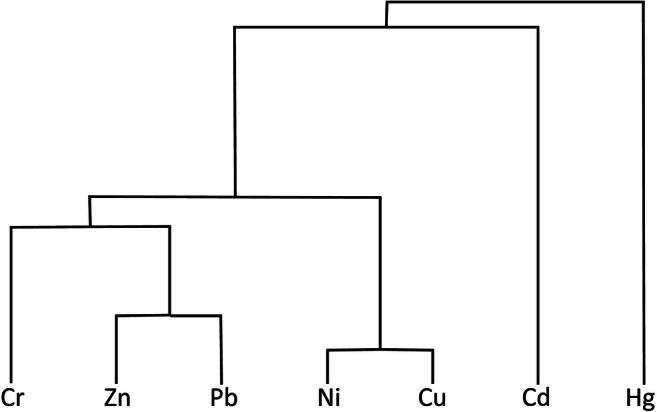
Results of cluster analysis, using Ward’s method, for data normalised to the background values

PCA results indicate that the three principal components account for 80% of variability (Table 8). The influence of elements on the components is also the basis for distinguishing groups of metals with similar characteristics. These groups are distinguished on the variability of elements, not their content. Therefore, one group can contain metals whose levels are similar to the background levels as well as metals whose levels considerably exceed the background levels. A similar variability of the levels of these metals is of key importance as it can suggest a similar origin (e.g. anthropogenic, or from rocks containing a considerable amount of these metals). The first component is mostly influenced by Cu, Pb, Zn and Ni, while the second one—by Cd, Cr and Hg (Fig. 4).

**Table 8:** Fragment of the matrix of eigenvectors with four principal components (italics indicate statistically important impact)

	Component I	Component II	Component III	Component IV
Cd	− 0.490	− *0.660*	0.272	− 0.483
Cr	− 0.352	− *0.716*	− *0.590*	0.070
Cu	− *0.876*	0.183	− 0.150	− 0.105
Hg	− 0.352	− *0.716*	− *0.590*	0.070
Ni	− *0.605*	− 0.533	0.325	0.477
Pb	− *0.912*	0.230	− 0.011	− 0.011
Zn	− *0.866*	0.159	0.106	0.025
Variation percentage	51.166	23.573	8.188	6.838
Total variation (%)	51.166	74.7	83	89.8

CA results show the existence of five separate statistical groups:

1. Ni, Cu—within the background levels or slightly exceeding them

2. Zn, Pb—two to three times as high as the background (more than twice as high on average)

3. Cr—four to five times as high as the background (more than 3.5 times on average)

4. Cd—over six times as high as the background and at the same time showing little variability with regard to content levels

5. Hg—on average twice as high as the background but having the greatest variability of content levels which can exceed the background the most—up to 27 times as high as the geochemical background

### Health risk

The values of indices describing the risk of carcinogenic and non-carcinogenic effects on the health of 6-year-old children within the playgrounds under study are very low. In all the analysed cases, the value of the hazard index (*HI*) is significantly below the hazard threshold for each element. Similarly in the case of cancer risk range index (*CR*) whose value for each of the elements analysed was well below the lower risk threshold, i.e. 10^−6^. For all the elements under study, it was from 0 to 10^−10^. This value was slightly below 10^−6^ only for Cr, but even then, it was still below the risk threshold (Table 9).

**Table 9:** Variability of *HI* and *CR* indices within the playgrounds under study

	Cd	Cr	Cu	Hg	Ni	Pb	Zn
	*HI*						
Mean	0.0045	0.056	0.0003	0.0001	0.0005	0.0096	0.0002
Median	0.0041	0.055	0.0003	0.00005	0.0004	0.0080	0.0002
Max. value	0.008	0.15	0.001	0.001	0.001	0.04	0.0012
Standard deviation	0.0010	0.022	0.0002	0.0002	0.0002	0.0063	0.0002
Coefficient of variation	0.2314	0.396	0.5697	1.7511	0.3381	0.6524	0.7629
	*CR*						
Mean	2 × 10^−9^	5.3 × 10^−6^	0	0	4 × 10^−11^	0	0
Median	1.3 × 10^−9^	3.1 × 10^−6^	0	0	2 × 10^−11^	0	0
Max. value	9.8 × 10^−9^	6.9 × 10^−6^	0	0	3.6 × 10^−10^	0	0
Standard deviation	1.5 × 10^−9^	8.9 × 10^−6^	0	0	5 × 10^−11^	0	0
Coefficient of variation	0.811	1.69	0	0	1.27	0	0

For 12 samples examined, mainly those from sandpits, the cancer risk range indices are below the 10^−6^ threshold. The other samples are below the less strict threshold at 10^−4^, and exceed the 10^−6^ threshold only slightly. This means that all the sites investigated are safe from the threat of non-carcinogenic and carcinogenic effects (Table 9).

The mean values of the hazard index are well below the health risk threshold and range from 0.0001 for Hg to 0.056 for Cr. The standard deviation of *HI* is small and shows that in all the cases analysed, the hazard index is below the hazard threshold, and there is no risk of non-carcinogenic health effects.

*CR* for Cu, Hg, Pb and Zn is zero, with an accuracy of 10^−15^. The highest *CR* occurs for chromium and its mean value is 5.3 × 10^−6^ ± 8.9 × 10^−6^. The variability index is 1.69, which means that *CR* is highly variable for this element. Ni is also characterised by a high variability, but the *CR* values for Ni are extremely low and do not exceed 3.6 × 10^−10^.

**Fig. 5:**
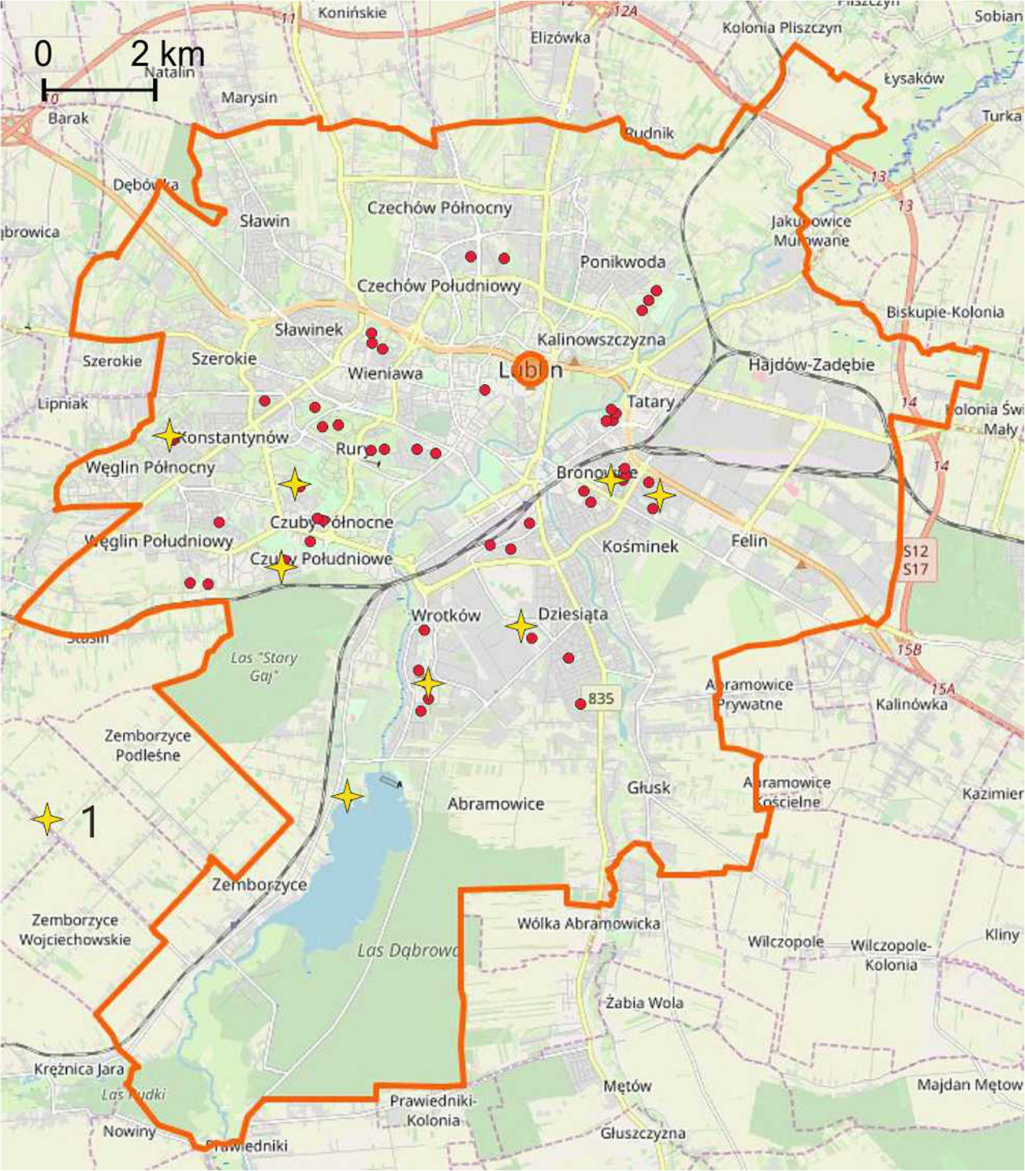
Playgrounds with the highest CR_tot_ index (1) (background map source: Open Street Map)

For the total *CR*, the mean is 5.3 × 10^−6^, the median is 3.1 × 10^−6^, and standard deviation is 8.9 × 10^−6^. The maximum value of *CR*_*tot*_ for the analysed samples is 6.97 × 10^−5^, which means that the sample with the highest degree of pollution with metals is still within the safety limit of 10^−6^–10^−4^. The variability index for *CR*_*tot*_ is 1.69. Most playgrounds with the highest *CR*_*tot*_ values are located in the eastern and south-eastern, i.e. the most industrialised, part of the city (Fig. 5).

## Discussion

A direct comparison of the obtained study results with data presented by other authors is difficult due to the different methods used to determine concentrations and examine the levels of metals in different granulometric fractions. However, it can be concluded that clearly greater amounts of Cd and Cr occurred in Lublin than in other cities, Cu and Ni levels were similar to the levels provided by other authors, while Pb and Zn levels were slightly lower. Similarly to the results of earlier studies, the levels of heavy metals in the sandpits were clearly lower than in the soil of the playgrounds in Lublin (Table 10).

**Table 10:** Levels of heavy metals in the soil and sandpits in playgrounds (mg kg^-1^)

	Cd	Cr	Cu	Ni	Pb	Zn
Soils—this study	4,6	192.2	19.2	11.7	47.4	90.3
Soils (under swings)—this study	5.1	209.3	17.5	13.8	40.2	97.5
Sandboxes—this study	4.5	177.8	11.4	13.6	32.0	52.6
Sandboxes1 (Kicińska 2018)	0.04	49	64	36	87	189
Sandboxes^2^ (Jasiewicz et al. 2009)	0.08	2.3	1.3	1.7	13.7	66
Soils^2^ (Nieć et al. 2013)	0.2–3.2	–	–	–	77–115	81–521
Sandy soils^3^ (De Miguel et al. 2007)	0.14	17	14	5.7	22	50
Soils^4^—urban parks (Kicińska 2016)	–	–	–	–	71–341	38–244
Sandboxes^4^—urban parks (Kicińska 2016)	–	–	–	–	28–36	17–103
Soils^5^ (Verla et al. 2015)	0.7	12.8	26.5	20.3	6.0	62.5

^1^< 63 μm, ^2^unknown fraction, ^3^< 100 μm, ^4^< 2000 μm, ^5^< 200 μm

A direct comparison of the identified health hazard with the results of studies conducted in other cities is not easy either due to the use of different methodologies to assess the concentration of metals, the fraction in which the concentration was determined, and the indices adopted in the calculations (exposure duration, body weight etc.). For Lublin, in all the analysed samples, the *HI* value is well below the risk threshold for non-carcinogenic effects on health. As it was stated earlier (Table 3), soils of Lublin are not polluted and the health risk caused by heavy metals is low.

A comparison with the results for Madrid shows that the health hazard is lower in the case of copper, nickel, zinc and lead (Table 11). In the case of cadmium, the value of this index is higher, but still well below the lower hazard threshold. The *HI* value for chromium is twice as high in Lublin than in Madrid and as much as four times as high as in the health resorts in southern Poland. The threats related to the presence of chromium in the soil of playgrounds were highlighted by Kicińska et al. (2017). The *HI* for playgrounds in health resorts in southern Poland is higher for Ni, Zn, Cd, comparable for Cu, while the hazard index for Cr and Pb is higher in Lublin (Table 11).

**Table 11:** Comparison of *HI* values in playgrounds for selected locations

Site	Cd	Cr	Cu	Ni	Pb	Zn
Madryd (De Miguel et al. 2007)	1 × 10^−3^	2.7 × 10^−2^	1.4 × 10^−3^	1.5 × 10^−3^	3 × 10^−2^	7.1 × 10^−4^
Spas in S Poland (Kicińska 2018)	6.3 × 10^−3^	1.4 × 10^−2^	3.4 × 10^−4^	9.6 × 10^−4^	5.2 × 10^−3^	4.0 × 10^−4^
Lublin (this work)	4.5 × 10^−3^	5.6 × 10^−2^	3 × 10^−4^	5 × 10^−4^	9.6 × 10^−3^	2 × 10^−4^

Carcinogenic and non-carcinogenic elements can be absorbed by the organism through ingestion, inhalation and skin contact. In all the cases analysed in the study, the ingestion of contaminated material was the key factor influencing the possible health hazard. The same conclusions were reached by Kicińska (2018) who studied playgrounds in health resorts in southern Poland. In Lublin, the mean value of *D*_*ing*_ is the highest for Cr (1.5 × 10^−3^) and the lowest for Hg (3.96 × 10^−8^). The highest mean value of *D*_*inh*_ was 4.2 × 10^−9^ for Cr and 1.1 × 10^−12^ for Hg, while that of *D*_*derm*_ was 4.1 × 10^−7^ for Cr and 1.1 × 10^−10^ for Hg. The other mean values of indices were between the mean values for Cr and mean values for Hg (Table 12). In the analysed situation, the ingestion of contaminated soil or sand from a sandpit occurs through the swallowing of the polluted material itself, not while eating food, as is most often the case with agricultural soil. It should be concluded, therefore, that the absorption of pollutants by children is even lower for the areas under study than what can be inferred from the theoretical calculations. The ingestion of polluted soil is rather accidental and sporadic although the amount of the material absorbed on a single occasion can be significant in some cases. Therefore, it should be concluded that the ingestion of contaminated soil by persons staying in playgrounds is not hazardous to health, in terms of the occurrence of carcinogenic and non-carcinogenic effects. At the same time, the pollution of soil with heavy metals does not always translate to a direct health hazard identified according to the accepted methodology of risk assessment because the harmfulness of the metals under study varies. In some cases, even if the concentration of a metal is considerably higher than the geochemical background, it does not lead to significant potential health effects. However, this problem undoubtedly requires further research on the influence of a long-lasting supply of small doses of heavy metals to the human body.

**Table 12:** Maximum and mean values of components of the hazard index: *D*_*ing*_ *D*_*inh*_ and *D*_*derm*_

	Cd	Cr	Cu	Hg	Ni	Pb	Zn
Mean value *D*_*ing*_	3.5 × 10^−6^	1.5 × 10^−3^	1.35 × 10^−5^	3.96 × 10^−8^	9.6 × 10^−6^	3.3 × 10^−5^	6.9 × 10^−5^
Max. value *D*_*ing*_	6.3 × 10^−6^	4 × 10^−3^	4.3 × 10^−5^	3.9 × 10^−7^	2.2 × 10^−5^	1.3 × 10^−4^	3 × 10^−4^
Mean value *D*_*inh*_	9.7 × 10^−11^	4.2 × 10^−9^	3.8 × 10^−10^	1.1 × 10^−12^	2.7 × 10^−10^	9.3 × 10^−10^	1.9 × 10^−9^
Max. value *D*_*inh*_	1.8 × 10^−10^	1.1 × 10^−8^	1.2 × 10^−9^	1.1 × 10^−11^	6.4 × 10^−10^	3.8 × 10^−9^	9.6 × 10^−9^
Mean value *D*_*derm*_	9.7 × 10^−9^	4.1 × 10^−7^	3.7 × 10^−8^	1.1 × 10^−10^	2.6 × 10^−8^	9.3 × 10^−8^	1.9 × 10^−7^
Max. value *D*_*derm*_	1.77 × 10^−8^	1.1 × 10^−6^	1.2 × 10^−7^	1.09 × 10^−9^	6.4 × 10^−8^	3.86 × 10^−7^	9.6 × 10^−7^

Elements whose levels are higher than the geochemical background, such as Cd, Hg, Cr, Zn and Pb, are of anthropogenic origin and the intensity of their supply varies, while Ni and Cu are mostly of natural origin. Pb and Zn are characterised by similar levels in relation to the background as well as similar variability, which suggests they are of mixed origin—anthropogenic and natural. Cd and Cr also exceed the geochemical background by a similar margin and have a similar variability, and can thus be regarded as elements of anthropogenic origin. Hg is a special element whose levels in most samples are similar to the natural levels but, in the case of a few playgrounds, exceed the background by a very high margin as a result of anthropogenic supply. In Poland, the main source of Hg emissions into the atmosphere is fuel combustion (63%), production of construction materials (26%), metallurgy (5%), chemical industry (4%) and waste combustion (2%) (Hławiczka and Fudała 2003). In Lublin, the location of sites most highly polluted with Hg to the highest degree indicates pollution caused by Hg emissions into the atmosphere as a result of fuel combustion in private homes.

Thus, the anthropogenic pollution of the soil in playgrounds seems to be a result of atmospheric deposition. The burning of fuels (coal, wood and other fuels) in private homes is the main source of metals discharged into the atmosphere in Lublin. In the eastern and south-eastern part of the city, the impact of industry and services can also be a contributing factor. The mean concentrations of the metals under study in playgrounds, normalised to the background values, are in the following order: Cd > Cr > Pb(Zn) > Hg > Ni > Cu. The order is slightly different than in the case of street dust (Zn > Cd > Cu > Cr > Pb > Ni), which may indicate that, in general, street dust is not a significant source of pollution of the soil in playgrounds (Zgłobicki et al. 2018). Locally, however, it can cause increased levels of heavy metals in the air and soil, particularly in the case of elements such as Cu and Zn. High concentrations in the soil in playgrounds were found in a few cases in the vicinity of streets with a high intensity of traffic (Fig. 4). The spatial distribution of the playgrounds with the highest concentrations of metals does not indicate any significant patterns and clear correlation with the city’s functional zones. This may indicate a significant role of local factors influencing atmospheric circulation such as topographic location, housing development patterns etc. Plak (2018) arrived at similar conclusions and noted that, in the case of the residential zones of Lublin, Toruń and Kraków, it is difficult to indicate one source of pollution with heavy metals.

## Conclusions

The studied metals supplied to the soil in the playgrounds were of varying origin. Cu and Ni can be regarded as elements of natural origin, Pb and Zn—of mixed origin, and Cd, Cr and Hg—of anthropogenic origin. In the case of the latter, some playgrounds showed a very intensive influence of human activity, while in other playgrounds, this influence was distinctly smaller.

Cd was the element with the highest degree of enrichment in relation to the natural levels (5 times) while a slightly smaller degree of enrichment was found for Cr (3 times). The geochemical background was exceeded most (26 times) in a single sample in the case of Hg. The values of geochemical indices show that the environment of playgrounds is polluted with Cd, in a few cases also with Hg and Cr. The highest mean concentrations occurred in samples collected from beneath the swings (Cd, Cr, Ni and Zn) or from the soil (Cu, Hg and Pb). The differences between the particular kinds of samples were not large; however, they were the largest for Hg and the smallest for Cu and Zn.

The values of indices describing the risk of carcinogenic and non-carcinogenic effects on health within the playgrounds under study are very low. Cr is the element posing the greatest threat. Also in this case, however, the health hazard indices are below the permitted limits. The ingestion of material containing metals was the key factor influencing the possibility of health effects, while inhalation and direct skin contact were clearly less significant (the volume of supply was smaller by several orders of magnitude).

The spatial distribution of playgrounds with the highest degree of pollution with heavy metals and the highest health hazard does not show a clear pattern. Factors conducive to higher concentrations include location within industrial and service districts, old parts of the city where stoves are used for heating as well as the proximity of streets with a high intensity of traffic.
